# Degradation and Stabilization of Polymer Materials

**DOI:** 10.3390/polym15234519

**Published:** 2023-11-24

**Authors:** Branka Mušič, Andrijana Sever Škapin

**Affiliations:** Slovenian National Building and Civil Engineering Institute, Dimičeva ulica 12, 1000 Ljubljana, Slovenia; andrijana.skapin@zag.si

## 1. Introduction

The growing awareness of the consequences of climate change has prompted the formulation of policies and regulations to foster sustainability. This global concern has had a far-reaching impact, influencing change and innovation in various sectors, including the plastics industry [1]. Researchers in this field face a myriad of challenges. They are engaged in several efforts, including (i) a substantial research and development endeavor that is directed towards the creation of sustainable and environmentally friendly materials, including recycled materials and biodegradable/bio-based plastics delivered from renewable resources. These materials are favored due to their reduced carbon footprint and lower environmental impact [2]. (ii) Research into the stabilization and degradation of plastic materials, where understanding the environmental implications of plastics is vital, as plastics affect the environment at all stages of their life cycle, from production to disposal. Researchers focus on assessing the environmental impact of plastics on terrestrial and aquatic ecosystems and studying the processes of plastic stabilization and degradation [3]. (iii) The development and use of novel technologies and analytical methods to determine the stability and degradation of polymeric materials [4,5,6].

In the field of plastics, the development of new polymer materials and methods that address environmental and sustainability concerns is being promoted. Novel materials are engineered to be more environmentally friendly, but their stabilization and tendency to degrade require careful assessment of their performance in various environmental conditions, including exposure to factors such as UV radiation, microorganisms, increased humidity, and temperature stress [7]. Plastics that enter natural environments during or after their lifetime decompose into microplastics and even nanoplastics, with diverse consequences for ecosystems and living organisms [8,9,10]. It is thus imperative to thoroughly investigate the full range of impacts of microplastics and nanoplastics on the environment. Scientists are encouraged to conduct extensive research in all relevant fields, and the general public should be informed about the potential negative consequences of the accumulation of plastics in the environment [11,12].

Given these challenges and ongoing research, the Special Issue “Degradation and Stabilization of Polymer Materials” presents ten articles by experts addressing various aspects and challenges related to (bio)plastic materials and their interaction with the environment. The articles explore research and development progress in the realm of (bio)polymers, encompassing their stabilization and degradation, as well as the formation and decomposition of microplastics and their environmental impact. The contributions are underpinned by significant innovations in characterization methods and techniques, offering a comprehensive perspective on this important field. A summary of the articles is provided below.

## 2. An Overview of Published Articles

Some polymers are susceptible to biodegradation in the natural environment by microorganisms such as bacteria and fungi or small organisms [13] such as earthworms. The Special Issue includes one review article by Khaldoon et al. (contribution 1) that highlighted the important contribution of earthworms to plastic degradation, connected issues and challenges, and their ability to ingest smaller plastic particles known as microplastics. Due to sustainable development, the terrestrial ecosystem receives increasing amounts of biodegradable and non-biodegradable plastic waste and microplastics. However, according to current studies, the biodegradability rate of this biodegradable plastic under natural conditions is not as high as regulated laboratory testing predicts. This article reviewed existing studies and through critical analysis revealed that earthworms can play an important role in the biodegradation of plastics and explained the challenges in this process. The toxicity and complexity of the plastic material, environmental factors such as temperature and soil moisture content, microbial population, and feeding method have been shown to have a significant effect on the biodegradation of plastics by earthworms, so further studies are needed to understand these factors in order to find suitable general conditions that can be achieved in the natural environment for the successful involvement of earthworms in the degradation of plastics. The authors warn, however, that the consequences of these processes could have side effects, such as the transfer of microplastics to the soil profile and groundwater and the absorption of microplastics in plant roots because earthworms grind microplastics to smaller sizes, which can not only cause groundwater pollution but also harm plants and human health by entering the food chain. Based on this review article, challenges in using earthworms for plastic degradation are expected to be mostly related to the toxicity and complexity of the plastic material, as well as environmental factors such as soil moisture content and soil temperature, microbial population, and feeding pattern.

The authors of contribution 2 also dealt with environmentally friendly polymers. Two water-soluble antibacterial agents were synthesized by starting the polymerization of isonitrile monomers with linear and star palladium catalysts, respectively, followed by quaternization with N,N-dimethyloctylamine. They presented an efficient strategy for the synthesis of polyisocyanide quaternary ammonium salts with a novel star-shaped structure. Due to the new structure, increased cation density, and improved water solubility, the prepared quaternary ammonium salts of star polyisocyanide show excellent antibacterial properties against *Escherichia coli* (*E. coli*) and *Staphylococcus aureus* (*S. aureus*) and with a hyperbranched structure of more peripheral alkyl chains. More terminal cations and higher positive charge density facilitate the attraction and lysis of bacteria. Polymeric quaternary ammonium salts are considered to be one of the most promising materials for antibacterial efficacy, and therefore, they will be the object of further research.

A promising material is also the polymeric film from the article “Degradation of P(3HB-co-4HB) Films in Simulated Body Fluids” (contribution 3) where the authors considered the stability of polymer films from a completely different, biomedical point of view. A new model for the preparation of biodegradable PHA copolymer films was used and the biodegradability of various PHA copolymers was evaluated and their biomedical applicability discussed. Two copolymers of 3-hydroxybutyrate and 4-hydroxybutyrate were prepared by growing thermophilic plants—P(3HB-co-36 mol.% 4HB) and P(3HB-co-66 mol.% 4HB)—the bacterial strain Aneurinibacillus sp. H1, and investigated degradability in simulated body fluids. Both showed faster weight loss in synthetic gastric juice and artificial intestinal fluid than the simple P3HB homopolymer. Synthetic gastric juice proved to be the most aggressive environment due to its low pH, resulting in the greatest weight loss of both copolymers. Most likely, this weight loss cannot be attributed to enzymatic hydrolysis. The authors state that the rate of biodegradation and concomitant biocompatibility are key factors in the selection of in vivo intestinal applications. Regarding the degradation rate of the copolymer, these materials appear to be very promising substitutes for other investigated materials such as PLA or PCL [14,15]. The results of this study confirm that there is a wide range of potential biomedical applications for the PHA family of polymers with specific degradation rate requirements that can be covered by adjusting the appropriate monomer composition. There are still many economic challenges and limitations in the biotechnological production of PHA, which are mainly influenced by the relatively high production costs and the difficulty of the extraction steps compared to other biopolymers. Nevertheless, many research groups are currently investigating new production approaches and cultivation strategies to increase the competitiveness of these highly promising biopolymers [16,17].

Yang, Du, and Liu also worked on a biodegradable polymer material, polylactic acid (PLA), and developed a novel environmentally friendly covalent organic framework/PLA composite material (contribution 4). A new high-strength, thermally stable, and degradable covalent organic framework (COF)-modified polylactic acid fiber (PLA) material (COF-PLA) was constructed for reinforcing the PLA material for environmentally friendly sand barriers. The micrographs, structure, thermal stability, and photodegradation products of COF-PLA were investigated. The results of this study indicated that the COF material was compatible with PLA and that the COF-PLA material took on the advantages of the COF by having a more regular arrangement, smoother surface, and smaller size, and it was more thermostable than PLA alone. After photodegradation, the COF-PLA material can produce melamine molecules that can neutralize the lactic acid and CO_2_ produced by PLA, which can maintain the acid–base balance in sandy soil and is beneficial to plant growth. Therefore, the degradation of COF-PLA does not cause pollution, making it a promising sand-control material that will continue to gain interest.

In order to minimize microplastic pollution, the group of Budhiraja et al. presented the magnetic extraction of tire wear (contribution 5). This work offers a rapid and low-cost solution for the separation of microplastics (MPs), whereby magnets are used to separate MPs from complex environmental matrices by magnetizing the hydrophobic surface of the MP. In this scientific research, they synthesized a hydrophobic Fe-silane-based nanocomposite (Fe@SiO_2_/MDOS), where the pristine and weathered polyethylene (PE) and tire wear particles (TWPs) were used. The weathering of MPs was performed in an accelerated weathering chamber. The chemical properties and morphology of the Fe@SiO_2_/MDOS, PE, and TWPs were confirmed via FTIR and SEM, respectively, and the thermal properties were evaluated using TGA. Using 1.00 mg of Fe@SiO_2_/MDOS nanocomposite, 2.00 mg of pristine and weathered PE were extracted from freshwater, whereas using the same amount of the nanocomposite, 7.92 mg of pristine TWPs and 6.87 mg of weathered TWPs were extracted. With this work, they provided a feasible strategy for the magnetic extraction of pristine and weathered PE and TWPs from freshwater using an iron-silane-based Fe@SiO_2_/MDOS nanocomposite. The magnetic separation technique can be used to remove MPs from the environment, which is simple, economical, and less time-consuming. Further work in magnetic separation toward large-scale applications could be made possible by the construction of a novel, inexpensive magnetic carrier media and separation devices.

Accelerated weathering of polyethylene microplastics was also considered by Budhiraja et al. (contribution 6). They determined the adsorption of organic pollutants onto weathered polyethylene MPs and their interaction, and demonstrated a change in the behavior of degraded MPs in the environment, i.e., their adsorption, degradation, toxicity, etc. In this study, a laboratory-accelerated weathering experiment was carried out with a virgin PE film and an oxidative degradable PE (OXO-PE) film, i.e., PE modified by the addition of a pro-oxidant catalyst. The differences caused by the degradation of PE and OXO-PE were assessed through FTIR spectroscopy, and the hydrophilic nature of the weathered samples was confirmed via contact angle measurements, TGA thermal analysis, and SEM morphology studies. Also, the adsorption of two model organic pollutants, triclosan and methylparaben (MeP), onto weathered and virgin PE was analyzed with GC-PID. The results also clearly show that OXO degradable plastics represent a fast route to MP degradation and may lead to the increased adsorption of pollutants. Both the effect of weathering on the adsorption of organic pollutants and the interaction between organic pollutants adsorbing onto MPs are highly relevant to actual MP pollution in the environment, where MPs are exposed to weathering conditions and mixtures of organic pollutants. Further research is suggested to study the combined toxic effect of weathered MPs (containing additives) and multiple coexisting pollutants on marine organisms.

Exposure to high temperatures can cause the degradation of polymers. This may result in chain scission, cross-linking, or the release of volatile compounds [18]. Thermal stability is a crucial consideration in many polymer applications. In contribution 7, the main influences, namely heat and moisture stress, that cause damage to switchgear components made of composite epoxy insulating materials are presented. This article deals with the question of the degradation and lifetime of epoxy thermoset resin. Insulation failure of composite epoxy insulation materials in distribution switchgear under the stress of heat and moisture is one of the leading causes of damage to components of such installations. They performed material accelerated aging experiments under three conditions: 75 °C and 95% relative humidity (RH), 85 °C and 95% RH, and 95 °C and 95% RH. The mechanical, thermal, chemical, and microstructural properties of the material were investigated. Based on the data, tensile strength and ester carbonyl bond (C=O) absorption in infrared spectra were chosen as failure criteria. At the failure points, the ester C=O absorption decreased to ~28% and the tensile strength decreased to 50%. The ATR-FTIR spectroscopy was used to monitor the degradation, and a lifetime prediction model was developed that can be used to estimate the lifetime of epoxy composites at different temperatures and high RH (95%). The degradation mechanism was identified as the hydrolysis of ester bonds in epoxy resin into organic acids and alcohols at high humidity and high temperature, disrupting the epoxy resin networks and weakening their mechanical strength. Meanwhile, the Ca^2+^ in fillers reacted with the carboxylic acids produced by the degradation of the epoxy resin ester bond to form carboxylates, which destroyed the resin–filler interface and increased the material hydrophilicity. The authors proposed the application of ATR-FTIR spectroscopy to easily monitor the degradation of the epoxy composite system of electrical insulation.

The choice of preparation and stabilization methods and additives used depends on the type of polymer, its intended use, and the environmental conditions it will encounter. Appropriate testing and quality control are also essential to ensure that the stabilization methods chosen effectively protect the polymer material during its intended lifetime, and to monitor the environmental impact of the (bio-based) plastic thus prepared. The three articles in this Special Issue deal with the development of methods for tracking/determining the stability and degradation of polymer materials. Al Khulaifi et al. have investigated the thermal depolymerization of a polymer in real-time (contribution 8), while Beiras et al. and Kuznetsova et al. have presented tools and methods to assess the degradability of plastics in seawater (contributions 9 and 10). In contribution 8, the isothermal decomposition of poly(methyl methacrylate) (PMMA) synthesized by the radical route of methyl methacrylate in the presence of azobisisobutyronitrile as the initiator was carried out and monitored with the DART-Tof-MS technique at different temperatures. For better understanding, they also used NMR, TGA, MS analysis, and size-exclusion chromatography analysis. DART mass spectrometry was successfully applied to study the isothermal decomposition of PMMA. It was found that the non-isothermal decomposition of PMMA studied with TGA shows two steps of weight loss. The isothermal decomposition of the polymer with the DART-Tof-MS method revealed only one weight loss stage. However, DART mass spectrometry was successfully applied to study the isothermal decomposition of PMMA.

Also of great importance is contribution 9, in which a practical tool for the assessment of polymer biodegradability in marine environments was presented, which can guide the development of truly biodegradable plastics. The alternative plastics labeled as compostable are replacing polyolefins. By fitting experimental data to a non-linear logistic model, the ultimate biodegradability can be calculated without taking into account the incubation time. Whereas the commercial products show negligible or very low marine biodegradability, one of the novel materials exceeds the 20% biodegradation threshold relative to fully marine biodegradable PHB after 28 days. Because of its short duration and environmental relevancy, this presented method is useful to classify commercial plastic products according to their potential biodegradability in marine conditions and to guide the development of novel polymeric materials intended to rapidly degrade in the sea. In most trials, the experimental data fitted the non-linear logistic model well. It was found that for the evaluation of thin films, micronization pretreatment is not always necessary, whereas for the evaluation of more compact materials, micronization is essential to reduce the particle size to at least less than 1 mm. Furthermore, it has been proven that below 250 µm, different fractions behave similarly and do not accelerate the rate of biodegradation with decreasing size. Further research could yield interesting results where the test could be further accelerated by increasing the density of marine heterotrophic microorganisms in the incubation vessels.

Kuznetsova, Shtykov, and Timerbaev (contribution 10) assessed the behavior of plastics in seawater using mass spectrometry which enables us to use reliable analytical tools to unravel the transformations of primary plastics exposed to the marine environment. The authors evaluated the performance of the isotope ratio mass spectrometry (IRMS) technique for identifying the origin of polymers and monitoring the compositional alterations due to its chemical degradation. The carbon and hydrogen isotope measurements in natural abundance have revealed that biopolymers incline to substantial chemical transformation upon prolonged exposure to seawater and sunlight irradiation. To assess the seawater-mediated aging that leads to the release of micro/nano fragments from plastic products, they propose the use of microfiltration as a front end to IRMS. The fragmentation of plastics was recorded, and it was found that the rate and extent of disintegration varied significantly for different classes of polymers. Another impact of plastics on the environment is that toxic metals are adsorbed on their surface from the seashore water. They addressed this issue by using inductively coupled mass spectrometry after nitric acid leaching. IRMS proved useful as a ‘one-stone-three-birds’ tool for the characterization of plastic polymers in a seawater environment. The method was shown to be capable of (i) acquiring reliable IR data that help with the identification of polymer debris contaminating seawater, (ii) differentiating between petroleum- and plant-derived polymers, and (iii) monitoring their compositional alterations due to chemical degradation in seawater. This study measured the degradation degree of different types of polymers in seawater, filling a knowledge gap on plastic pollution and providing a useful methodology and important reference data for future research. Forthcoming research will be directed towards revealing the structural changes in plastics during degradation as well as the structure of disintegrated fragments.

## 3. Conclusions

This Special Issue covers the crucial topics of polymeric material degradation and stabilization, both of which are paramount in the polymer industry as well as for the preservation of our environment. The continuous emergence of new polymeric materials that are intended for end-users and may enter the environment as waste after use makes it essential to understand how they are affected by different environmental conditions.

Following the presentation of the collected articles, it is evident that the Special Issue “Degradation and Stabilization of Polymer Materials” comprehensively encompasses the multidisciplinary nature of the field. The ten articles featured in the issue span a wide spectrum, including the development of novel materials, innovative testing and analytical methods, applications in biomedicine and electronics, as well as their implications in both marine and terrestrial environments. This multidisciplinary approach has the potential to significantly influence the plastics industry and its environmental footprint.

We are grateful for the contributions in the form of original articles in the Special Issue, as they have greatly enriched our understanding of these critical subjects.
